# Ethyl 2-[2-(2,4-diphenyl-3-aza­bicyclo­[3.3.1]nonan-9-yl­idene)hydrazin-1-yl]-4-methyl-1,3-thia­zole-5-carboxyl­ate di­methyl­formamide monosolvate

**DOI:** 10.1107/S1600536813033540

**Published:** 2013-12-18

**Authors:** S. Jothivel, S. Kabilan

**Affiliations:** a Department of Chemistry, Annamalai University, Annamalainagar 608 002, Chidambaram, Tamil Nadu, India

## Abstract

In the title mol­ecule, C_27_H_30_N_4_O_2_S·C_3_H_7_NO, the fused piperidine and cyclo­hexane rings adopt a twin chair conformation and the phenyl groups occupy equatorial sites. The phenyl rings make a dihedral angle of 40.74 (2)°. In the crystal, the di­methyl­formamide solvent mol­ecule is connected to the main mol­ecule by an N—H⋯O hydrogen bond. An additional N—H⋯O hydrogen bond connects mol­ecules into chains along [100]. Pairs of weak C—H⋯O hydrogen bonds connect inversion-related chains. The ethyl group was refined as disordered over two sets of sites with an occupancy ratio of 0.660 (17):0.340 (17).

## Related literature   

For the biological activity of related structures, see: Ramachandran *et al.* (2009 ▶); Hutchinson *et al.* (2002 ▶); Bondock *et al.* (2007 ▶). For bicyclic compounds, see: Jeyaraman & Avila (1981 ▶).
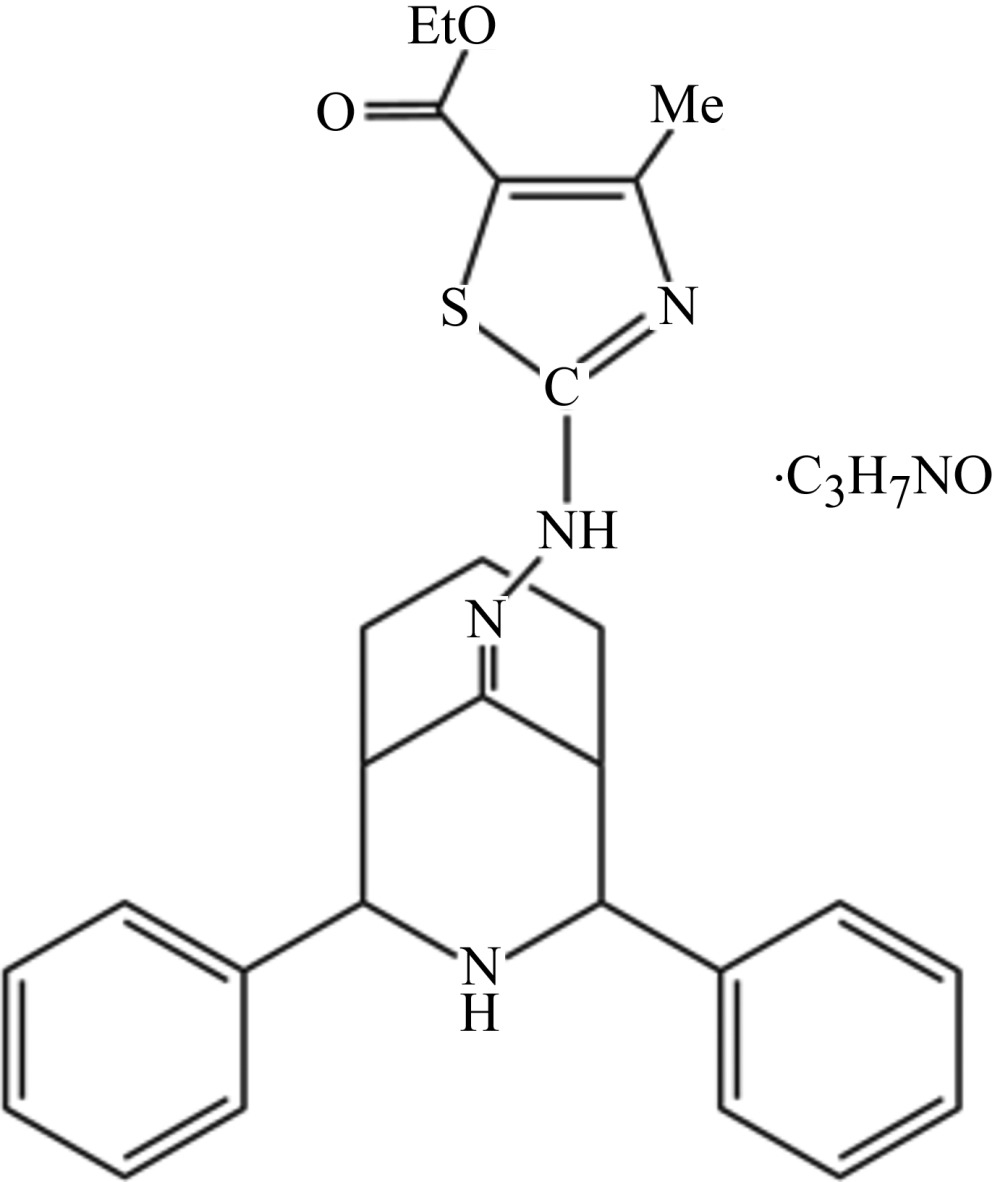


## Experimental   

### 

#### Crystal data  

C_27_H_30_N_4_O_2_S·C_3_H_7_NO
*M* *_r_* = 547.71
Monoclinic, 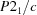

*a* = 12.700 (5) Å
*b* = 19.427 (5) Å
*c* = 13.203 (5) Å
β = 115.249 (5)°
*V* = 2946.3 (18) Å^3^
*Z* = 4
Mo *K*α radiation
μ = 0.15 mm^−1^
*T* = 293 K
0.35 × 0.35 × 0.30 mm

#### Data collection  

Bruker Kappa APEXII CCD diffractometer
Absorption correction: multi-scan (*SADABS*; Bruker, 1999 ▶) *T*_min_ = 0.937, *T*_max_ = 0.965
25815 measured reflections
5179 independent reflections
3606 reflections with *I* > 2σ(*I*)
*R*_int_ = 0.032

#### Refinement  

*R*[*F* ^2^ > 2σ(*F* ^2^)] = 0.055
*wR*(*F* ^2^) = 0.154
*S* = 1.01
5179 reflections
379 parameters
40 restraints
H atoms treated by a mixture of independent and constrained refinement
Δρ_max_ = 0.56 e Å^−3^
Δρ_min_ = −0.37 e Å^−3^

### 

Data collection: *APEX2* (Bruker, 2004 ▶); cell refinement: *APEX2* and *SAINT* (Bruker, 2004 ▶); data reduction: *SAINT* and *XPREP* (Bruker, 2004 ▶; program(s) used to solve structure: *SIR92* (Altomare *et al.*, 1993 ▶); program(s) used to refine structure: *SHELXL97* (Sheldrick, 2008 ▶); molecular graphics: *SHELXTL* (Sheldrick, 2008 ▶) and *PLATON* (Spek, 2009 ▶); software used to prepare material for publication: *SHELXL97*.

## Supplementary Material

Crystal structure: contains datablock(s) I, 1. DOI: 10.1107/S1600536813033540/lh5671sup1.cif

Structure factors: contains datablock(s) I. DOI: 10.1107/S1600536813033540/lh5671Isup2.hkl

Supporting information file. DOI: 10.1107/S1600536813033540/lh5671Isup3.cdx

Supporting information file. DOI: 10.1107/S1600536813033540/lh5671Isup4.cml

Additional supporting information:  crystallographic information; 3D view; checkCIF report

## Figures and Tables

**Table 1:** Hydrogen-bond geometry (Å, °)

*D*—H⋯*A*	*D*—H	H⋯*A*	*D*⋯*A*	*D*—H⋯*A*
C14—H14⋯O1^i^	0.93	2.41	3.284 (4)	156
N1—H1*A*⋯O1^ii^	0.84 (2)	2.59 (2)	3.380 (4)	157 (2)
N3—H3*A*⋯O3	0.85 (2)	1.99 (2)	2.843 (4)	173 (3)

Symmetry codes: (i) 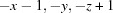; (ii) 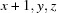.

**Fig. 1.**
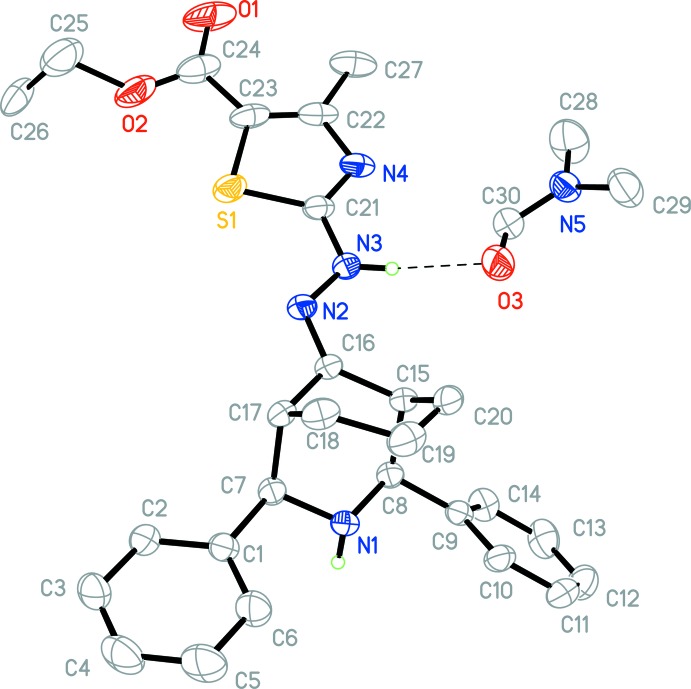
The molecular structure of the title compound, showing 30% probability displacement ellipsoids.

**Fig. 2.**
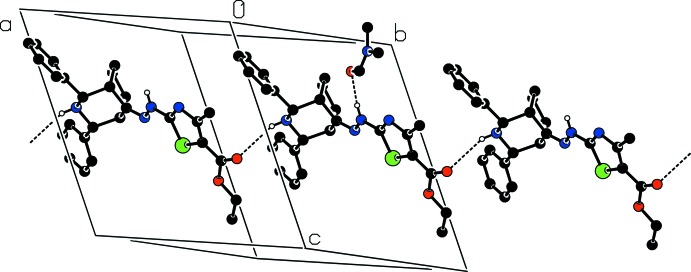
Part of the crystal structure with hydrogen bonds shown as dashed lines.

## supplementary crystallographic information

### 1. Comment

Thiazoles are an interesting unit in medicinal chemistry and are responsible for numerous pharmacological and biological properties (Hutchinson *et al.* 2002; Bondock *et al.*, 2007; Ramachandran *et al.*, 2009). This has piqued our interest in the synthesis of thiazole containing compounds. The importance of bicyclic compounds as intermediates in the synthesis of a several physiologically active compounds have been reviewed by Jeyaraman & Avila (1981). Moreover, these bridged bicyclic compounds exhibit twin chair, chair–boat or twin boat conformations and possess interesting stereochemistries. In order to investigate the change in molecular conformation of the piperidine and cyclohexane rings, the X-ray structure determination of the title compound was carried out. The six–membered heterocyclic piperidine ring (Fig. 1) adopts the expected chair conformation. The two phenyl rings form a dihedral angle of 40.74 (2)°. In the crystal the dimethylformamide solvent molecule is connected to the main molecule by an N—H···O hydrogen bond. An additional N—H···O hydrogen bond connects molecules into chains along [100] (Fig. 2). Weak C—H···O hydrogen bonds connect pairs of inversion related chains. The ethyl group was refined as disordered over two sets of sites with a 0.660 (17): 0.340 (17) ratio of occupancies.

### 2. Experimental

To a boiling solution of the bicyclic thiosemicarbazone (0.01 mol) in ethanolic–chloroform (1:1 / v:v), ethyl-2-chloroacetoacetate(0.01 mol), sodium acetate trihydrate (0.02 mol) and a few drops of acetic acid were added and refluxed for about 5–6 h. After the completion of reaction, excess of solvent was removed under reduced pressure and poured into water. After work–up, the solid was separated and purified by column chromatography using benzene–ethyl acetate (9:1 / v:v) as eluent on neutral alumina. Colourless crystals were grown by slow evaporation method using dimethylformamide as the solvent.

### 3. Refinement

H atoms bonded to C atoms were included in calculated positions with C—H = 0.93-0.98Å and included in the refinement with U_iso_(H) = 1.2U_eq_(C) or 1.5U_eq_(C_methyl_). H atoms bonded to N atoms were refined independently with isotropic displacment parameters.

### Crystal data

**Table:**

C_27_H_30_N_4_O_2_S·C_3_H_7_NO	*F*(000) = 1168
*M**_r_* = 547.71	*D*_x_ = 1.235 Mg m^−^^3^
Monoclinic, *P*2_1_/*c*	Mo *K*α radiation, λ = 0.71073 Å
Hall symbol: -P 2ybc	Cell parameters from 6962 reflections
*a* = 12.700 (5) Å	θ = 2.1–22.4°
*b* = 19.427 (5) Å	µ = 0.15 mm^−^^1^
*c* = 13.203 (5) Å	*T* = 293 K
β = 115.249 (5)°	Block, colourless
*V* = 2946.3 (18) Å^3^	0.35 × 0.35 × 0.30 mm
*Z* = 4	

### Data collection

**Table:**

Bruker Kappa APEXII CCD diffractometer	5179 independent reflections
Radiation source: fine-focus sealed tube	3606 reflections with *I* > 2σ(*I*)
Graphite monochromator	*R*_int_ = 0.032
ω and φ sacn scans	θ_max_ = 25.0°, θ_min_ = 2.0°
Absorption correction: multi-scan (*SADABS*; Bruker, 1999)	*h* = −15→15
*T*_min_ = 0.937, *T*_max_ = 0.965	*k* = −20→23
25815 measured reflections	*l* = −15→15

### Refinement

**Table:**

Refinement on *F*^2^	Primary atom site location: structure-invariant direct methods
Least-squares matrix: full	Secondary atom site location: difference Fourier map
*R*[*F*^2^ > 2σ(*F*^2^)] = 0.055	Hydrogen site location: inferred from neighbouring sites
*wR*(*F*^2^) = 0.154	H atoms treated by a mixture of independent and constrained refinement
*S* = 1.01	*w* = 1/[σ^2^(*F*_o_^2^) + (0.0579*P*)^2^ + 2.3626*P*] where *P* = (*F*_o_^2^ + 2*F*_c_^2^)/3
5179 reflections	(Δ/σ)_max_ = 0.001
379 parameters	Δρ_max_ = 0.56 e Å^−^^3^
40 restraints	Δρ_min_ = −0.37 e Å^−^^3^

### Special details

**Table:**

Geometry. All e.s.d.'s (except the e.s.d. in the dihedral angle between two l.s. planes) are estimated using the full covariance matrix. The cell e.s.d.'s are taken into account individually in the estimation of e.s.d.'s in distances, angles and torsion angles; correlations between e.s.d.'s in cell parameters are only used when they are defined by crystal symmetry. An approximate (isotropic) treatment of cell e.s.d.'s is used for estimating e.s.d.'s involving l.s. planes.
Refinement. Refinement of *F*^2^ against ALL reflections. The weighted *R*-factor *wR* and goodness of fit *S* are based on *F*^2^, conventional *R*-factors *R* are based on *F*, with *F* set to zero for negative *F*^2^. The threshold expression of *F*^2^ > σ(*F*^2^) is used only for calculating *R*-factors(gt) *etc*. and is not relevant to the choice of reflections for refinement. *R*-factors based on *F*^2^ are statistically about twice as large as those based on *F*, and *R*- factors based on ALL data will be even larger.

### Fractional atomic coordinates and isotropic or equivalent isotropic displacement parameters (Å^2^)

**Table:**

	*x*	*y*	*z*	*U*_iso_*/*U*_eq_	Occ. (<1)
C1	0.0735 (2)	0.24044 (14)	0.5466 (2)	0.0499 (7)	
C2	0.0996 (3)	0.27051 (16)	0.6495 (3)	0.0647 (8)	
H2	0.0572	0.2579	0.6892	0.078*	
C3	0.1867 (3)	0.31863 (18)	0.6946 (3)	0.0815 (11)	
H3	0.2018	0.3387	0.7634	0.098*	
C4	0.2508 (3)	0.33680 (19)	0.6382 (4)	0.0891 (12)	
H4	0.3109	0.3686	0.6690	0.107*	
C5	0.2264 (3)	0.3081 (2)	0.5363 (4)	0.0902 (12)	
H5	0.2692	0.3211	0.4972	0.108*	
C6	0.1386 (3)	0.25983 (18)	0.4907 (3)	0.0706 (9)	
H6	0.1234	0.2403	0.4215	0.085*	
C7	−0.0232 (2)	0.18797 (13)	0.5011 (2)	0.0450 (6)	
H7	−0.0184	0.1591	0.5638	0.054*	
C8	−0.0971 (2)	0.08953 (13)	0.3738 (2)	0.0445 (6)	
H8	−0.0939	0.0613	0.4365	0.053*	
C9	−0.0710 (2)	0.04318 (13)	0.2953 (2)	0.0452 (6)	
C10	−0.0280 (2)	0.06844 (15)	0.2225 (2)	0.0546 (7)	
H10	−0.0154	0.1155	0.2208	0.066*	
C11	−0.0035 (3)	0.02520 (19)	0.1523 (3)	0.0673 (9)	
H11	0.0247	0.0434	0.1036	0.081*	
C12	−0.0204 (3)	−0.0437 (2)	0.1539 (3)	0.0750 (10)	
H12	−0.0027	−0.0727	0.1073	0.090*	
C13	−0.0633 (3)	−0.07009 (17)	0.2242 (3)	0.0743 (10)	
H13	−0.0757	−0.1172	0.2248	0.089*	
C14	−0.0887 (3)	−0.02709 (15)	0.2951 (3)	0.0613 (8)	
H14	−0.1180	−0.0458	0.3427	0.074*	
C15	−0.2198 (2)	0.12259 (15)	0.3180 (2)	0.0490 (7)	
H15	−0.2783	0.0858	0.2944	0.059*	
C16	−0.2341 (2)	0.16510 (14)	0.4064 (2)	0.0472 (6)	
C17	−0.1453 (2)	0.22093 (14)	0.4488 (2)	0.0495 (7)	
H17	−0.1569	0.2461	0.5076	0.059*	
C18	−0.1687 (3)	0.27046 (16)	0.3508 (3)	0.0626 (8)	
H18A	−0.2426	0.2933	0.3319	0.075*	
H18B	−0.1086	0.3055	0.3749	0.075*	
C19	−0.1718 (3)	0.23575 (17)	0.2467 (3)	0.0665 (9)	
H19A	−0.0928	0.2250	0.2583	0.080*	
H19B	−0.2043	0.2675	0.1841	0.080*	
C20	−0.2433 (2)	0.17010 (17)	0.2176 (2)	0.0625 (8)	
H20A	−0.2274	0.1449	0.1623	0.075*	
H20B	−0.3252	0.1823	0.1837	0.075*	
C21	−0.4752 (2)	0.10489 (15)	0.4414 (2)	0.0518 (7)	
C22	−0.6309 (2)	0.06611 (18)	0.4541 (3)	0.0641 (9)	
C23	−0.5903 (3)	0.10747 (18)	0.5450 (3)	0.0670 (9)	
C24	−0.6432 (4)	0.1188 (2)	0.6213 (4)	0.0859 (12)	
C27	−0.7378 (3)	0.0215 (2)	0.4136 (4)	0.0902 (12)	
H27A	−0.7461	−0.0024	0.3470	0.135*	
H27B	−0.8051	0.0498	0.3974	0.135*	
H27C	−0.7305	−0.0113	0.4706	0.135*	
C28	−0.7208 (4)	−0.0655 (2)	0.1020 (4)	0.1106 (15)	
H28A	−0.7792	−0.0754	0.0281	0.166*	
H28B	−0.7567	−0.0450	0.1455	0.166*	
H28C	−0.6827	−0.1074	0.1371	0.166*	
C29	−0.6557 (3)	0.0071 (2)	−0.0138 (3)	0.0943 (12)	
H29C	−0.6091	0.0475	−0.0054	0.141*	
H29A	−0.7365	0.0185	−0.0551	0.141*	
H29B	−0.6344	−0.0275	−0.0534	0.141*	
C30	−0.5483 (3)	0.00313 (18)	0.1851 (3)	0.0675 (8)	
H30	−0.5396	−0.0152	0.2533	0.081*	
N1	−0.00799 (18)	0.14310 (11)	0.41935 (18)	0.0453 (5)	
N2	−0.31082 (18)	0.16070 (12)	0.44462 (18)	0.0512 (6)	
N3	−0.39631 (19)	0.11185 (14)	0.3983 (2)	0.0567 (6)	
N4	−0.56551 (19)	0.06438 (13)	0.3952 (2)	0.0583 (6)	
N5	−0.6363 (2)	−0.01846 (13)	0.0948 (2)	0.0637 (7)	
O1	−0.7381 (2)	0.09901 (17)	0.6088 (3)	0.1174 (11)	
O2	−0.5722 (3)	0.15330 (17)	0.7104 (2)	0.1002 (9)	
O3	−0.4768 (2)	0.04548 (16)	0.1866 (2)	0.0917 (8)	
S1	−0.46094 (7)	0.14717 (5)	0.56068 (7)	0.0648 (3)	
C25	−0.588 (2)	0.1963 (16)	0.8026 (13)	0.120 (6)	0.340 (17)
H25A	−0.5743	0.2446	0.7939	0.144*	0.340 (17)
H25B	−0.6672	0.1914	0.7946	0.144*	0.340 (17)
C26	−0.5059 (18)	0.1725 (11)	0.9147 (13)	0.091 (5)	0.340 (17)
H26A	−0.5358	0.1840	0.9682	0.137*	0.340 (17)
H26B	−0.4319	0.1945	0.9354	0.137*	0.340 (17)
H26C	−0.4965	0.1235	0.9135	0.137*	0.340 (17)
C25'	−0.6228 (7)	0.1578 (6)	0.7918 (6)	0.085 (2)	0.660 (17)
H25C	−0.6998	0.1783	0.7589	0.103*	0.660 (17)
H25D	−0.6268	0.1130	0.8225	0.103*	0.660 (17)
C26'	−0.5381 (12)	0.2028 (9)	0.8765 (14)	0.140 (5)	0.660 (17)
H26D	−0.5628	0.2118	0.9345	0.210*	0.660 (17)
H26E	−0.5326	0.2455	0.8423	0.210*	0.660 (17)
H26F	−0.4633	0.1808	0.9081	0.210*	0.660 (17)
H1A	0.0570 (18)	0.1231 (13)	0.451 (2)	0.051 (8)*	
H3A	−0.415 (2)	0.0916 (13)	0.3356 (17)	0.051 (8)*	

### Atomic displacement parameters (Å^2^)

**Table:**

	*U*^11^	*U*^22^	*U*^33^	*U*^12^	*U*^13^	*U*^23^
C1	0.0418 (14)	0.0513 (16)	0.0553 (16)	−0.0015 (12)	0.0194 (12)	−0.0048 (13)
C2	0.0544 (17)	0.070 (2)	0.073 (2)	−0.0053 (15)	0.0304 (16)	−0.0243 (17)
C3	0.064 (2)	0.075 (2)	0.096 (3)	−0.0101 (18)	0.026 (2)	−0.040 (2)
C4	0.065 (2)	0.070 (2)	0.118 (3)	−0.0231 (18)	0.025 (2)	−0.023 (2)
C5	0.078 (2)	0.093 (3)	0.107 (3)	−0.034 (2)	0.047 (2)	−0.001 (2)
C6	0.069 (2)	0.080 (2)	0.068 (2)	−0.0245 (17)	0.0339 (17)	−0.0077 (17)
C7	0.0438 (14)	0.0508 (15)	0.0434 (14)	−0.0053 (12)	0.0215 (12)	−0.0018 (12)
C8	0.0411 (13)	0.0510 (15)	0.0436 (14)	−0.0048 (12)	0.0203 (11)	−0.0011 (12)
C9	0.0353 (13)	0.0489 (16)	0.0490 (15)	−0.0025 (11)	0.0158 (11)	−0.0046 (12)
C10	0.0482 (16)	0.0595 (17)	0.0653 (18)	−0.0076 (13)	0.0331 (14)	−0.0098 (15)
C11	0.0583 (18)	0.083 (2)	0.072 (2)	0.0003 (16)	0.0382 (17)	−0.0126 (18)
C12	0.077 (2)	0.079 (3)	0.070 (2)	0.0138 (19)	0.0332 (19)	−0.0153 (19)
C13	0.091 (2)	0.0468 (18)	0.071 (2)	0.0058 (17)	0.0211 (19)	−0.0102 (16)
C14	0.0672 (19)	0.0564 (18)	0.0578 (18)	−0.0036 (15)	0.0242 (15)	0.0037 (15)
C15	0.0359 (13)	0.0662 (18)	0.0490 (15)	−0.0060 (12)	0.0220 (12)	−0.0080 (13)
C16	0.0380 (13)	0.0634 (17)	0.0438 (14)	0.0022 (12)	0.0210 (12)	−0.0036 (13)
C17	0.0449 (14)	0.0598 (17)	0.0525 (16)	−0.0029 (13)	0.0290 (13)	−0.0102 (13)
C18	0.0561 (17)	0.0620 (19)	0.075 (2)	0.0106 (15)	0.0330 (16)	0.0056 (16)
C19	0.0674 (19)	0.078 (2)	0.0617 (19)	0.0187 (17)	0.0348 (16)	0.0212 (17)
C20	0.0484 (16)	0.093 (2)	0.0455 (16)	0.0174 (16)	0.0193 (13)	−0.0005 (16)
C21	0.0392 (14)	0.0680 (18)	0.0523 (16)	0.0086 (13)	0.0235 (13)	0.0115 (14)
C22	0.0426 (16)	0.077 (2)	0.080 (2)	0.0187 (15)	0.0330 (16)	0.0376 (19)
C23	0.0543 (18)	0.085 (2)	0.079 (2)	0.0233 (17)	0.0450 (17)	0.037 (2)
C24	0.080 (3)	0.114 (3)	0.086 (3)	0.041 (2)	0.057 (2)	0.050 (2)
C27	0.0486 (18)	0.098 (3)	0.130 (3)	0.0033 (18)	0.044 (2)	0.033 (2)
C28	0.109 (3)	0.117 (3)	0.105 (3)	−0.050 (3)	0.045 (3)	−0.007 (3)
C29	0.074 (2)	0.134 (4)	0.063 (2)	−0.019 (2)	0.0183 (18)	0.007 (2)
C30	0.061 (2)	0.078 (2)	0.060 (2)	0.0036 (18)	0.0231 (17)	−0.0044 (17)
N1	0.0351 (11)	0.0515 (13)	0.0495 (13)	−0.0012 (10)	0.0182 (10)	−0.0055 (11)
N2	0.0390 (12)	0.0712 (15)	0.0486 (13)	−0.0025 (11)	0.0238 (10)	−0.0022 (12)
N3	0.0427 (13)	0.0850 (18)	0.0492 (14)	−0.0094 (12)	0.0262 (11)	−0.0104 (13)
N4	0.0361 (12)	0.0736 (16)	0.0659 (15)	0.0040 (11)	0.0224 (11)	0.0138 (13)
N5	0.0546 (15)	0.0708 (17)	0.0600 (16)	−0.0065 (13)	0.0190 (13)	−0.0008 (13)
O1	0.0866 (19)	0.164 (3)	0.145 (3)	0.0323 (19)	0.0910 (19)	0.058 (2)
O2	0.096 (2)	0.148 (3)	0.0854 (18)	0.0435 (19)	0.0665 (17)	0.0204 (18)
O3	0.0672 (15)	0.135 (2)	0.0749 (16)	−0.0336 (16)	0.0326 (13)	−0.0304 (15)
S1	0.0582 (5)	0.0880 (6)	0.0615 (5)	0.0065 (4)	0.0383 (4)	0.0060 (4)
C25	0.119 (13)	0.152 (14)	0.117 (11)	0.027 (11)	0.077 (10)	−0.002 (12)
C26	0.121 (11)	0.099 (11)	0.079 (8)	0.025 (8)	0.067 (8)	−0.005 (7)
C25'	0.096 (5)	0.096 (6)	0.099 (4)	−0.001 (4)	0.075 (4)	−0.009 (4)
C26'	0.133 (11)	0.165 (11)	0.147 (12)	−0.027 (9)	0.083 (10)	−0.078 (9)

### Geometric parameters (Å, º)

**Table:**

C1—C6	1.375 (4)	C20—H20A	0.9700
C1—C2	1.383 (4)	C20—H20B	0.9700
C1—C7	1.509 (4)	C21—N4	1.308 (4)
C2—C3	1.375 (4)	C21—N3	1.354 (3)
C2—H2	0.9300	C21—S1	1.716 (3)
C3—C4	1.364 (5)	C22—C23	1.351 (5)
C3—H3	0.9300	C22—N4	1.358 (4)
C4—C5	1.364 (5)	C22—C27	1.503 (5)
C4—H4	0.9300	C23—C24	1.445 (5)
C5—C6	1.383 (5)	C23—S1	1.746 (3)
C5—H5	0.9300	C24—O1	1.208 (5)
C6—H6	0.9300	C24—O2	1.320 (5)
C7—N1	1.462 (3)	C27—H27A	0.9600
C7—C17	1.542 (4)	C27—H27B	0.9600
C7—H7	0.9800	C27—H27C	0.9600
C8—N1	1.464 (3)	C28—N5	1.443 (4)
C8—C9	1.512 (4)	C28—H28A	0.9600
C8—C15	1.551 (4)	C28—H28B	0.9600
C8—H8	0.9800	C28—H28C	0.9600
C9—C10	1.382 (4)	C29—N5	1.435 (4)
C9—C14	1.383 (4)	C29—H29C	0.9600
C10—C11	1.381 (4)	C29—H29A	0.9600
C10—H10	0.9300	C29—H29B	0.9600
C11—C12	1.357 (5)	C30—O3	1.219 (4)
C11—H11	0.9300	C30—N5	1.308 (4)
C12—C13	1.361 (5)	C30—H30	0.9300
C12—H12	0.9300	N1—H1A	0.844 (17)
C13—C14	1.392 (4)	N2—N3	1.374 (3)
C13—H13	0.9300	N3—H3A	0.853 (17)
C14—H14	0.9300	O2—C25'	1.472 (6)
C15—C16	1.502 (4)	O2—C25	1.559 (15)
C15—C20	1.536 (4)	C25—C26	1.474 (16)
C15—H15	0.9800	C25—H25A	0.9700
C16—N2	1.277 (3)	C25—H25B	0.9700
C16—C17	1.491 (4)	C26—H26A	0.9600
C17—C18	1.536 (4)	C26—H26B	0.9600
C17—H17	0.9800	C26—H26C	0.9600
C18—C19	1.517 (4)	C25'—C26'	1.465 (11)
C18—H18A	0.9700	C25'—H25C	0.9700
C18—H18B	0.9700	C25'—H25D	0.9700
C19—C20	1.517 (4)	C26'—H26D	0.9600
C19—H19A	0.9700	C26'—H26E	0.9600
C19—H19B	0.9700	C26'—H26F	0.9600
			
C6—C1—C2	117.7 (3)	C19—C20—H20A	108.7
C6—C1—C7	123.0 (3)	C15—C20—H20A	108.7
C2—C1—C7	119.3 (2)	C19—C20—H20B	108.7
C3—C2—C1	121.6 (3)	C15—C20—H20B	108.7
C3—C2—H2	119.2	H20A—C20—H20B	107.6
C1—C2—H2	119.2	N4—C21—N3	121.6 (3)
C4—C3—C2	119.8 (3)	N4—C21—S1	116.3 (2)
C4—C3—H3	120.1	N3—C21—S1	122.1 (2)
C2—C3—H3	120.1	C23—C22—N4	115.4 (3)
C3—C4—C5	119.7 (3)	C23—C22—C27	127.1 (3)
C3—C4—H4	120.1	N4—C22—C27	117.5 (3)
C5—C4—H4	120.1	C22—C23—C24	126.5 (3)
C4—C5—C6	120.6 (4)	C22—C23—S1	110.6 (2)
C4—C5—H5	119.7	C24—C23—S1	122.9 (3)
C6—C5—H5	119.7	O1—C24—O2	123.1 (4)
C1—C6—C5	120.6 (3)	O1—C24—C23	126.1 (5)
C1—C6—H6	119.7	O2—C24—C23	110.8 (4)
C5—C6—H6	119.7	C22—C27—H27A	109.5
N1—C7—C1	110.8 (2)	C22—C27—H27B	109.5
N1—C7—C17	110.0 (2)	H27A—C27—H27B	109.5
C1—C7—C17	112.9 (2)	C22—C27—H27C	109.5
N1—C7—H7	107.6	H27A—C27—H27C	109.5
C1—C7—H7	107.6	H27B—C27—H27C	109.5
C17—C7—H7	107.6	N5—C28—H28A	109.5
N1—C8—C9	110.3 (2)	N5—C28—H28B	109.5
N1—C8—C15	110.2 (2)	H28A—C28—H28B	109.5
C9—C8—C15	113.0 (2)	N5—C28—H28C	109.5
N1—C8—H8	107.7	H28A—C28—H28C	109.5
C9—C8—H8	107.7	H28B—C28—H28C	109.5
C15—C8—H8	107.7	N5—C29—H29C	109.5
C10—C9—C14	117.5 (3)	N5—C29—H29A	109.5
C10—C9—C8	122.1 (2)	H29C—C29—H29A	109.5
C14—C9—C8	120.4 (2)	N5—C29—H29B	109.5
C11—C10—C9	121.3 (3)	H29C—C29—H29B	109.5
C11—C10—H10	119.4	H29A—C29—H29B	109.5
C9—C10—H10	119.4	O3—C30—N5	124.9 (3)
C12—C11—C10	120.5 (3)	O3—C30—H30	117.6
C12—C11—H11	119.8	N5—C30—H30	117.6
C10—C11—H11	119.8	C7—N1—C8	113.88 (19)
C11—C12—C13	119.6 (3)	C7—N1—H1A	108.3 (19)
C11—C12—H12	120.2	C8—N1—H1A	107.3 (19)
C13—C12—H12	120.2	C16—N2—N3	117.5 (2)
C12—C13—C14	120.5 (3)	C21—N3—N2	117.9 (2)
C12—C13—H13	119.7	C21—N3—H3A	114.5 (19)
C14—C13—H13	119.7	N2—N3—H3A	126.0 (19)
C9—C14—C13	120.6 (3)	C21—N4—C22	110.3 (3)
C9—C14—H14	119.7	C30—N5—C29	121.0 (3)
C13—C14—H14	119.7	C30—N5—C28	120.9 (3)
C16—C15—C20	107.2 (2)	C29—N5—C28	118.0 (3)
C16—C15—C8	106.8 (2)	C24—O2—C25'	109.6 (4)
C20—C15—C8	116.4 (2)	C24—O2—C25	134.6 (12)
C16—C15—H15	108.7	C21—S1—C23	87.38 (16)
C20—C15—H15	108.7	C26—C25—O2	110.1 (13)
C8—C15—H15	108.7	C26—C25—H25A	109.6
N2—C16—C17	118.8 (2)	O2—C25—H25A	109.6
N2—C16—C15	129.5 (2)	C26—C25—H25B	109.6
C17—C16—C15	111.7 (2)	O2—C25—H25B	109.6
C16—C17—C18	107.2 (2)	H25A—C25—H25B	108.1
C16—C17—C7	108.7 (2)	C25—C26—H26A	109.5
C18—C17—C7	115.7 (2)	C25—C26—H26B	109.5
C16—C17—H17	108.4	H26A—C26—H26B	109.5
C18—C17—H17	108.4	C25—C26—H26C	109.5
C7—C17—H17	108.4	H26A—C26—H26C	109.5
C19—C18—C17	114.0 (2)	H26B—C26—H26C	109.5
C19—C18—H18A	108.8	C26'—C25'—O2	100.7 (9)
C17—C18—H18A	108.8	C26'—C25'—H25C	111.6
C19—C18—H18B	108.8	O2—C25'—H25C	111.6
C17—C18—H18B	108.8	C26'—C25'—H25D	111.6
H18A—C18—H18B	107.7	O2—C25'—H25D	111.6
C18—C19—C20	112.6 (2)	H25C—C25'—H25D	109.4
C18—C19—H19A	109.1	C25'—C26'—H26D	109.5
C20—C19—H19A	109.1	C25'—C26'—H26E	109.5
C18—C19—H19B	109.1	H26D—C26'—H26E	109.5
C20—C19—H19B	109.1	C25'—C26'—H26F	109.5
H19A—C19—H19B	107.8	H26D—C26'—H26F	109.5
C19—C20—C15	114.3 (2)	H26E—C26'—H26F	109.5
			
C6—C1—C2—C3	0.6 (5)	C7—C17—C18—C19	66.9 (3)
C7—C1—C2—C3	−179.9 (3)	C17—C18—C19—C20	45.7 (3)
C1—C2—C3—C4	−1.1 (5)	C18—C19—C20—C15	−45.1 (3)
C2—C3—C4—C5	1.3 (6)	C16—C15—C20—C19	53.1 (3)
C3—C4—C5—C6	−1.1 (6)	C8—C15—C20—C19	−66.4 (3)
C2—C1—C6—C5	−0.4 (5)	N4—C22—C23—C24	179.7 (3)
C7—C1—C6—C5	−179.9 (3)	C27—C22—C23—C24	−1.6 (5)
C4—C5—C6—C1	0.7 (6)	N4—C22—C23—S1	−0.4 (3)
C6—C1—C7—N1	22.4 (4)	C27—C22—C23—S1	178.2 (3)
C2—C1—C7—N1	−157.1 (3)	C22—C23—C24—O1	−8.7 (6)
C6—C1—C7—C17	−101.5 (3)	S1—C23—C24—O1	171.5 (3)
C2—C1—C7—C17	79.0 (3)	C22—C23—C24—O2	170.0 (3)
N1—C8—C9—C10	−39.0 (3)	S1—C23—C24—O2	−9.8 (4)
C15—C8—C9—C10	84.8 (3)	C1—C7—N1—C8	178.4 (2)
N1—C8—C9—C14	140.4 (3)	C17—C7—N1—C8	−56.0 (3)
C15—C8—C9—C14	−95.8 (3)	C9—C8—N1—C7	−176.8 (2)
C14—C9—C10—C11	−0.1 (4)	C15—C8—N1—C7	57.7 (3)
C8—C9—C10—C11	179.3 (3)	C17—C16—N2—N3	175.5 (2)
C9—C10—C11—C12	−0.5 (5)	C15—C16—N2—N3	−2.0 (4)
C10—C11—C12—C13	0.9 (5)	N4—C21—N3—N2	173.3 (2)
C11—C12—C13—C14	−0.7 (5)	S1—C21—N3—N2	−8.0 (4)
C10—C9—C14—C13	0.4 (4)	C16—N2—N3—C21	177.3 (3)
C8—C9—C14—C13	−179.1 (3)	N3—C21—N4—C22	179.8 (3)
C12—C13—C14—C9	0.0 (5)	S1—C21—N4—C22	1.0 (3)
N1—C8—C15—C16	−57.6 (3)	C23—C22—N4—C21	−0.3 (4)
C9—C8—C15—C16	178.4 (2)	C27—C22—N4—C21	−179.1 (3)
N1—C8—C15—C20	62.0 (3)	O3—C30—N5—C29	0.1 (5)
C9—C8—C15—C20	−61.9 (3)	O3—C30—N5—C28	−176.0 (4)
C20—C15—C16—N2	113.4 (3)	O1—C24—O2—C25'	4.6 (6)
C8—C15—C16—N2	−121.2 (3)	C23—C24—O2—C25'	−174.1 (5)
C20—C15—C16—C17	−64.3 (3)	O1—C24—O2—C25	−20.2 (15)
C8—C15—C16—C17	61.1 (3)	C23—C24—O2—C25	161.0 (14)
N2—C16—C17—C18	−113.0 (3)	N4—C21—S1—C23	−1.0 (2)
C15—C16—C17—C18	65.0 (3)	N3—C21—S1—C23	−179.8 (3)
N2—C16—C17—C7	121.3 (3)	C22—C23—S1—C21	0.8 (2)
C15—C16—C17—C7	−60.7 (3)	C24—C23—S1—C21	−179.4 (3)
N1—C7—C17—C16	55.5 (3)	C24—O2—C25—C26	128.4 (18)
C1—C7—C17—C16	179.9 (2)	C25'—O2—C25—C26	81 (3)
N1—C7—C17—C18	−65.1 (3)	C24—O2—C25'—C26'	−174.6 (10)
C1—C7—C17—C18	59.3 (3)	C25—O2—C25'—C26'	−28.6 (14)
C16—C17—C18—C19	−54.5 (3)		

### Hydrogen-bond geometry (Å, º)

**Table:**

*D*—H···*A*	*D*—H	H···*A*	*D*···*A*	*D*—H···*A*
C14—H14···O1^i^	0.93	2.41	3.284 (4)	156
N1—H1*A*···O1^ii^	0.84 (2)	2.59 (2)	3.380 (4)	157 (2)
N3—H3*A*···O3	0.85 (2)	1.99 (2)	2.843 (4)	173 (3)

Symmetry codes: (i) −*x*−1, −*y*, −*z*+1; (ii) *x*+1, *y*, *z*.
